# Transformation of Lignin Under Protection Strategies: Catalytic Oxidation and Depolymerization by Polyoxometalates Catalysts

**DOI:** 10.3390/polym16243480

**Published:** 2024-12-13

**Authors:** Xinyue Ma, Wenbiao Xu

**Affiliations:** 1 Faculty of Bioscience Engineering, Jilin Agricultural Science and Technology University, Hanlin Rond, Jilin City 132101, China; 2 College of Materials Science and Engineering, Beihua University, Jilin City 132013, China

**Keywords:** lignin, pretreatment, polyoxometalates, oxidative transformation, chemicals

## Abstract

The efficient utilization of lignin, a pivotal component of lignocellulosic biomass, is crucial for advancing sustainable biorefinery processes. However, optimizing lignin valorization remains challenging due to its intricate structure and susceptibility to undesirable reactions during processing. In this study, we delve into the impact of various pretreatment agents on birch lignin, aiming to enhance its catalytic oxidation and depolymerization under polyoxometalates (POMs) catalysis. Our results reveal that pretreatment with formaldehyde effectively safeguards aryl ether linkages in lignin, leading to a notable increase in aromatic compound yields under POMs catalysis. Furthermore, gel permeation chromatography (GPC) analysis underscores the inhibition of aryl ether linkage hydrolysis upon formaldehyde addition. Gas chromatography–mass spectrometry (GC–MS) analysis demonstrates that formaldehyde pretreatment boosts lignin monomer yield by 2 to 3 times compared to untreated lignin, underscoring the effectiveness of tailored pretreatment strategies. This research underscores the significance of adopting rational pretreatment methods to advance lignin valorization pathways catalyzed by POMs, thereby contributing to the evolution of sustainable biomass conversion technologies.

## 1. Introduction

As fossil fuel reserves dwindle, the global focus has shifted towards harnessing renewable biomass resources as substitutes [1]. Lignin, an abundantly occurring macromolecular polymer in biomass, constitutes approximately 15–40% of the dry mass of natural lignocellulose, ranking second only to cellulose. It plays a pivotal role in providing fundamental structural support to plant cell walls alongside cellulose and hemicellulose [2,3]. With its distinctive aromatic ring structure, lignin possesses diverse functional groups and chemical properties, offering vast potential in the realms of energy, chemicals, and materials [4]. Thus, it holds significant importance for promoting rational resource utilization and fostering sustainable development [5,6]. Despite its considerable promise, lignin’s structural complexity and the presence of recalcitrant chemical bonds present numerous unresolved challenges in selectively depolymerizing lignin into high-value chemicals [7]. Current transformation methods encompass pyrolysis, oxidative-reductive depolymerization, hydrogenolysis, microwave-assisted depolymerization, and biological depolymerization [8]. While these techniques facilitate effective lignin depolymerization, they encounter issues such as low conversion efficiency, propensity for intermediate product condensation, demanding reaction conditions, and challenges in product separation [9]. Therefore, further research is imperative to overcome these obstacles and advance lignin valorization [10,11,12,13].

The extraction of lignin is a crucial step for its effective utilization. Various pretreatment methods are employed to isolate lignin from lignocellulosic biomass, including acid, alkali, and organic solvent pretreatment [14]. Acid and alkali pretreatment procedures subject lignin to high temperatures and pressures, resulting in significant structural damage to most lignin molecules and the formation of recalcitrant carbon–carbon linkage structures [15]. Consequently, organic solvent pretreatment has emerged as the predominant method for lignin extraction in current research [16]. This method is considered more selective and milder compared to acid and alkali treatments, leading to less degradation of the lignin structure. The incorporation of protective agents during pretreatment effectively shields functional groups on the lignin side chains, thus inhibiting intermolecular condensation of lignin molecules and facilitating the enhancement of lignin depolymerization product yields [17]. In recent studies, protective agents have been shown to improve not only the yield but also the selectivity of the products, which is crucial for further applications of lignin depolymerization. Li et al. [18] conducted biomass pretreatment under acidic conditions, where the addition of formaldehyde solvent allowed formaldehyde molecules to undergo condensation reactions with the hydroxyl structures on the lignin side chains, forming stable ring structures and suppressing the generation of benzyl cations. This resulted in hydrogenolysis yields 3–7 times higher compared to lignin without protective agents. Lan et al. [19] investigated the impact of different diol protective agents during biomass extraction on the monomer yield of lignin after hydrogenolysis. They found that when acetaldehyde and propionaldehyde were used as protective agents, the monomer yields were 37 wt% and 42 wt%, respectively, comparable to yields obtained with formaldehyde as a protective agent, and exhibited favorable selectivity towards products. These findings emphasize the critical role of protective agents in improving both the yield and product distribution, which has been a limitation in earlier methods. Overall, the incorporation of protective agents during lignin pretreatment holds promise for enhancing depolymerization product yields by inhibiting intermolecular condensation [20,21].

Existing strategies for lignin depolymerization, particularly those employing protective agents in conjunction with noble metal catalyzed hydrogenolysis pathways, exhibit limitations such as low selectivity, high costs associated with noble metal catalysts, and the requirement for harsh reaction conditions [22,23,24,25]. In contrast, oxidative transformation pathways present several advantages, including milder reaction conditions, higher selectivity towards desired products, and potential utilization of more readily available and cost-effective catalysts [26,27,28]. Therefore, exploration of oxidative transformation routes for lignin valorization presented a promising strategy to address the limitations associated with conventional hydrogenolysis approaches [29,30,31,32]. 

Within this framework, our study aimed to validate the protective effects of various pretreatment agents, including formaldehyde, acetaldehyde, propionaldehyde, and ethylene glycol, on the hydroxyl groups at the side chain positions of lignin. We compared the yields of aromatic compounds obtained through oxidative depolymerization under different pretreatment conditions, optimized the reaction parameters, and assessed the effectiveness of these strategies across various lignin sources. Additionally, we employed lignin model compounds to elucidate the underlying reaction mechanisms, thereby establishing a foundation for the application of lignin protection strategies in catalytic oxidation processes for the synthesis of oxygen-containing functionalized aromatic compounds. The highlight of our work lies in our attempt to optimize the catalytic oxidation performance of lignin by protecting its side-chain hydroxyl groups with pretreatment reagents. Our study contributes to the advancement of lignin valorization by elucidating effective pretreatment strategies and reaction mechanisms, thus paving the way for the development of sustainable processes for the synthesis of oxygen-containing functionalized aromatic compounds.

## 2. Materials and Methods

### 2.1. Materials

Birch veneers were cut into small pieces and dried to constant weight. The dried birch wood pieces were then crushed using a grinder and sieved through a 60-mesh sieve. The sieved birch wood powder was placed in a planetary ball mill and ball-milled for 4 h. The resulting ball-milled birch wood powder was collected and set aside for later use. Other chemicals were purchased from Sigma and used directly without further treatment. 

### 2.2. Lignin Extraction

The dewaxed birch sawdust samples were ball-milled using a Retsch PM100 planetary ball mill (Retsch GmbH, Haan, Germany) equipped with a 250 mL ZrO_2_ jar and ZrO_2_ milling balls (5 × 15 mm and 10 × 5 mm). The milling process was conducted at a rotation speed of 450 rpm with a programmed cycle of 10 min of milling followed by a 15-min pause, for a total duration of 5 h. After ball-milling the crushed birch wood powder, the milled powder underwent a defatting process. The defatting procedure involved wrapping the birch wood powder in defatting filter paper and placing it into a Soxhlet extractor. Organic solvents benzene and ethanol (2:1 ratio) were added to the bottom of the flask. The Soxhlet extractor was placed in a fume hood and extracted for 6 h, ensuring extraction was conducted 4–6 times per hour. After extraction, the birch wood powder was dried in an oven for later use. To prevent the protective effects that novel green separation methods, such as deep eutectic solvents and ionic liquids, may have during the lignin separation process, we employed the traditional dioxane separation method. This approach enabled a comparative analysis of the structural characteristics and conversion performance of lignin under the influence of different protective agents. Lignin was extracted using an organic solvent method, following the procedure outlined by Lan et al. [19]: 50 g of ball-milled birch wood powder was placed in a three-necked flask, into which a mixture of 250 mL of 1,4-dioxane, 48 mL of formaldehyde (or acetaldehyde, propionaldehyde, or ethylene glycol), and 8.5 mL of hydrochloric acid (37 wt%) was added. The entire setup was placed in an oil bath and stirred, heated, and refluxed for 4 h. After the reaction, the mixture was allowed to cool to room temperature, followed by the addition of 16.8 g of sodium bicarbonate, and stirred for 30 min. The solid–liquid separation was performed using vacuum filtration, retaining the mixed liquid, which was washed twice. The filtrate was subjected to vacuum distillation in a water bath at 40 °C to obtain a concentrated liquid. Then, 150 mL of ethyl acetate was added to the concentrated liquid, and it was then dripped into 500 mL of *n*-hexane. After precipitation, the mixture was filtered, washed with 500 mL of distilled water, and dried. The resulting solid product was birch lignin.

### 2.3. Lignin Reaction with POMs

In a typical reaction, 0.25 g of H_3_PMo_12_O_40_ catalyst was combined with 0.25 g of lignin, with and without added protective agents (formaldehyde, acetaldehyde, propionaldehyde, and ethylene glycol), in a 10 mL high-pressure reactor. After adding 10 mL methanol/H_2_O (8:2, *v*:*v*) to the reactor, oxygen was introduced until the air inside the reactor was completely replaced. Oxygen was then introduced to the desired pressure, and the reaction temperature was set to 150 °C with a stirring rate of 500 rpm for 1 h. After the reaction was complete, the reactor was cooled to room temperature, and the product was filtered, rotary evaporated, and extracted to obtain the degraded lignin oil product. 

### 2.4. Depolymerized Products Analysis

For the analysis of the lignin bio-oil composition obtained from the experiments, an Agilent 6890N/5973i GC–MS system was utilized. The detection conditions were adapted from the reference [33]. The molecules were identified by comparing the mass spectrometry (MS) spectra with the database provided by the National Institute of Standards and Technology (NIST). Two-dimensional heteronuclear single quantum coherence nuclear magnetic resonance spectroscopy (2D HSQC NMR) (Bruker Avance II 500 MHz NMR, Karlsruhe, Germany) was employed to determine the structural characteristics of birch lignin extracted under different solvent pretreatment conditions, examining the influence of various solvents on lignin structural features. Experimental conditions followed the methods outlined in reference [34]. The experimental conditions for GPC (Agilent Infinity 1260 HPLC, Palo Alto, CA, USA) analysis were conducted following procedures outlined in the previous literature [35,36]. FT-IR (Tensor 27 Fourier transform infrared spectrometer, BRUKER, Karlsruhe, Germany) was utilized to determine the functional group characteristics present in lignin, aiming to analyze the effects of various organic solvents on the structure of birch lignin. The detailed experimental procedures follow the methods reported in the literature [37].

## 3. Results and Discussion

### 3.1. Analysis of Lignin Structure Under Different Pretreatment Conditions

**Figure 1:**
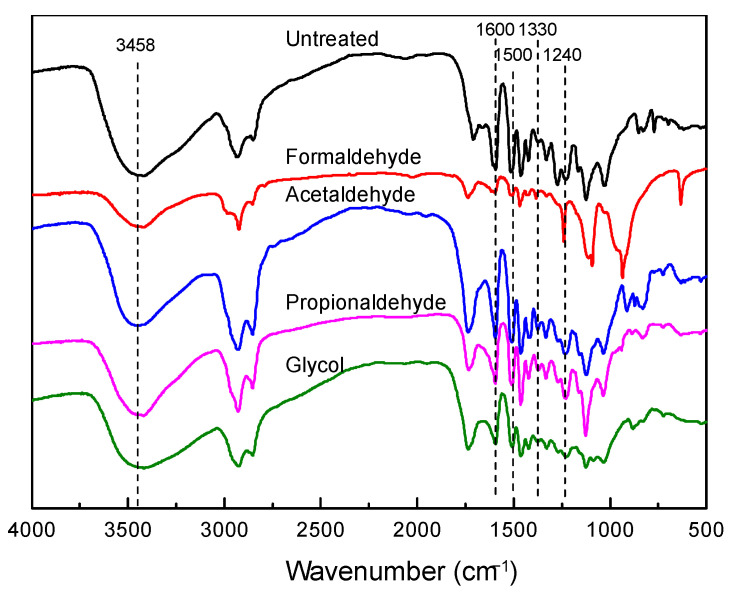
Infrared spectra of lignin without and with different protective agents.

Comparison analysis of the infrared spectra of lignin samples, with and without the addition of various protective agents, provides fundamental functional group information for these lignin variants. The infrared spectra of each sample are depicted in Figure 1, with the assignment of functional groups according to previous reports [38]. The infrared spectra obtained from the pretreated lignin primarily exhibit bands distributed in two major regions: 2800–3500 cm^−1^ and 600–1750 cm^−1^. Within the range of 3000–3500 cm^−1^, distinct stretching vibration peaks of hydroxyl O-H bonds are evident. Following the addition of protective agents, a notable decrease in the vibration of hydroxyl groups in lignin is observed, particularly pronounced in lignin treated with formaldehyde. This suggests that these small-molecule protective agents occupy the hydroxyl groups at the C_α_-C_β_ positions in lignin, forming active sites after hydrolysis under acidic conditions. Additionally, distinct peaks associated with methyl and methylene C-H stretching vibrations are observed at 2842–3000 cm^−1^ and 1460–1470 cm^−1^, respectively. Near 1700 cm^−1^, peaks corresponding to carbonyl groups (ketones and carboxylic acids) are observed. Peaks characteristic of two lignin aromatic rings appear near 1600 cm^−1^ and 1500 cm^−1^. Furthermore, a stretching vibration peak of lignin syringyl unit C–O bonds is observed at 1330 cm^−1^, while a stretching vibration peak of lignin guaiacyl unit C–O bonds appears at 1200 cm^−1^. These findings indicate that the addition of protective agents enhances the C–O absorption peaks at these two positions, forming stable six-membered ring structures, resulting in lignin producing more C–O–C bonds compared to those extracted without protective agents.

**Figure 2:**
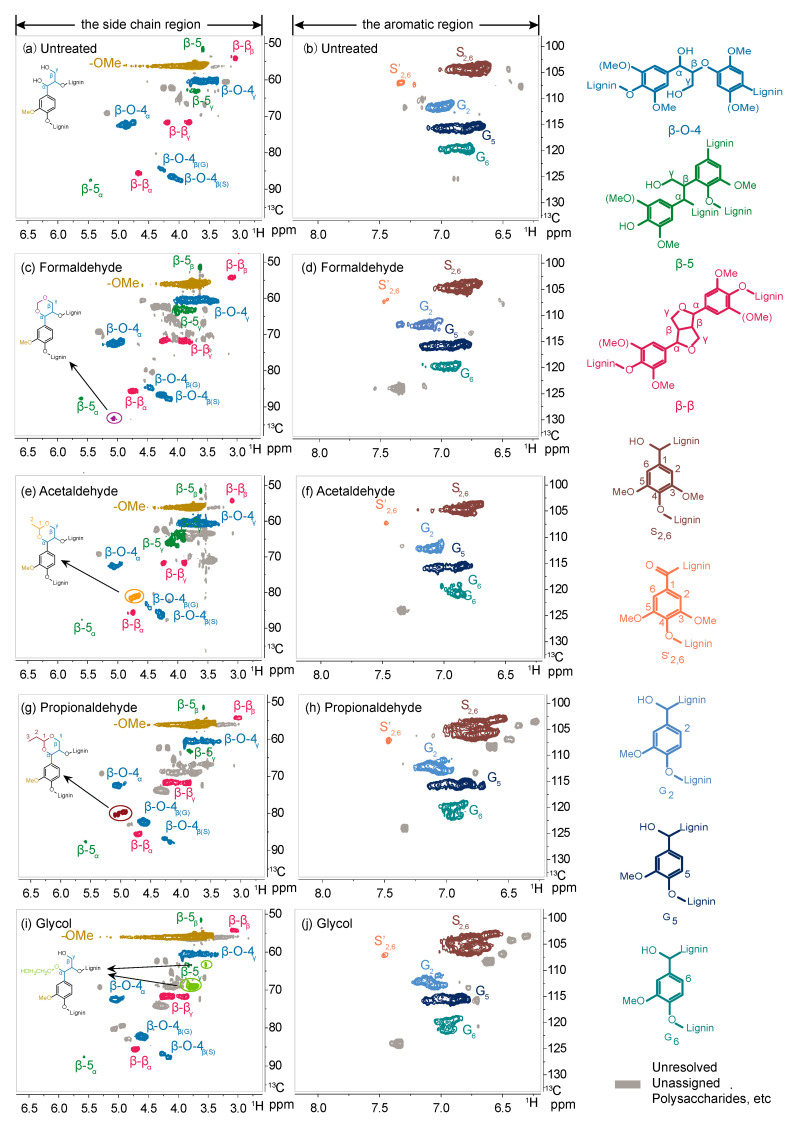
2D HSQC NMR spectra of organic solvent lignin extracted with different protective agents during the pretreatment process.

To further explore the influence of various solvents on the structural characteristics and linkages of lignin, lignin samples were characterized using 2D HSQC NMR spectroscopy. The side chain region mainly reflects the basic linkage modes of benzyl propane units in lignin, primarily including β-O-4, β-5, and β-β linkages. The aromatic region reflects primarily the three basic units (G, S, and H units) and end groups in lignin structure. During the reaction process, the addition of a suitable amount of hydrochloric acid induces dehydration of lignin C_α_-OH, generating an active site, which reacts with the benzene ring structure of lignin to form more resistant carbon–carbon bonds. This is also one of the reasons for the relatively low lignin yield. It can be observed from the spectrum that lignin extracted with organic solvents contains abundant β-O-4 linkages, indicating the presence of a large number of ether bonds in natural lignin structure. According to the lignin structural assignment in relevant literature [39,40], compared to lignin without added protective agents (Figure 2), lignin extracted with formaldehyde, acetaldehyde, and propionaldehyde exhibit new chemical shift signals corresponding to (δ_C_/δ_H_ 94/5.2 ppm), (δ_C_/δ_H_ 82/4.75 ppm), and (δ_C_/δ_H_ 82/4.75–5.0 ppm), respectively, indicating a higher content of β-O-4 linkages than lignin without protective agents. This is because the protective agents form a cyclic acetal protection structure with the lignin side chain, and in the absence of formaldehyde, no signals of cyclic acetal structures were observed. New chemical shift signals (δ_C_/δ_H_ 63/3.5 ppm and 70/3.75 ppm) appeared in lignin extracted with ethylene glycol, indicating the α-position etherification of β-5 and β-β linkages. This suggests that under organic solvent extraction, the β-β and β-5 structures are relatively stable. These results indicate that organic solvents react with lignin to preserve the natural ether bond structure of lignin as much as possible. Therefore, the use of organic solvent extraction for lignin separation from biomass is a promising approach. Organic solvents can preserve lignin’s more natural structure, providing a potentially effective method for lignin stabilization upgrading.

**Figure 3:**
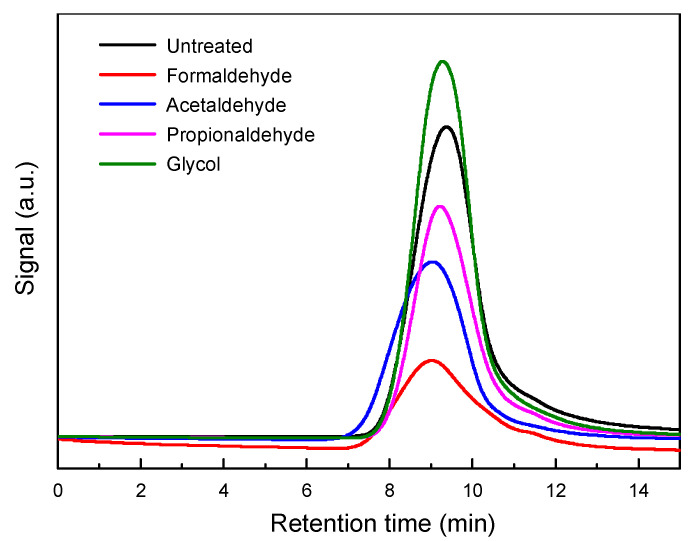
GPC of lignin with different stabilizers.

During the extraction of lignin, accompanied by partial hydrolysis and re-polymerization of lignin, GPC was employed to characterize the molecular weights of these lignin samples to explore the influence of different protective agent pretreatments on lignin’s molecular weight [41]. The results shown in Figure 3 display the weight-average molecular weight (Mw), number-average molecular weight (Mn), and polydispersity index of these lignin samples (shown in Table 1). Analysis revealed that the molecular weight of unstabilized lignin was relatively low, with Mw at 7714 g/mol and Mn at 3482 g/mol, significantly lower than that of the other four types of pretreated lignin. On one hand, during the extraction of lignin using organic solvent methods, some aryl ether bonds may undergo hydrolysis in acidic environments, leading to a decrease in lignin molecular weight. On the other hand, during the extraction of lignin, some lignin molecules may condense to form more resistant C–C bonds, resulting in changes in molecular weight during this process. Based on the GPC results, it can be inferred that the addition of stabilizers results in lignin with lower degrees of hydrolysis compared to those without stabilizers. Among them, the addition of formaldehyde leads to less hydrolysis of lignin, retaining more aryl ether bonds, thereby maintaining the chemical structure of lignin more intact, and similar effects are observed with other pretreatment agents. In summary, the addition of stabilizers preserves more of the original C–O bond structure of lignin, with the molecular weight being more influenced by the hydrolysis of aryl ether bonds during the extraction process and less affected by the formation of C–C bonds from lignin fragments.

### 3.2. Analysis of Depolymerized Products Under Different Pretreatment Conditions

**Figure 4:**
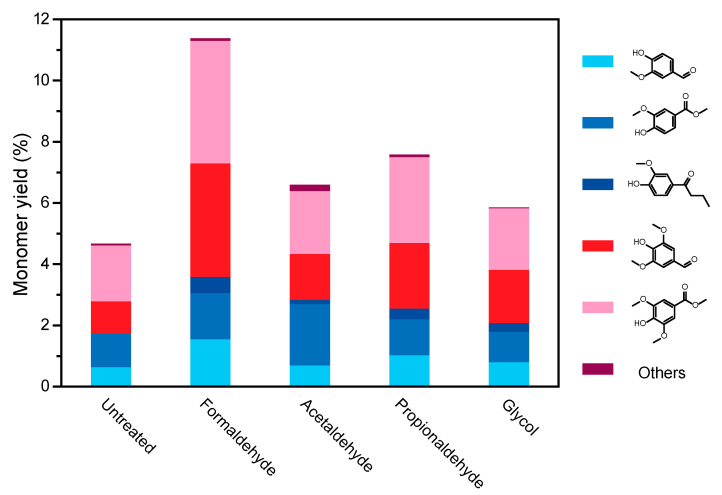
Distribution of products and yields of lignin oxygenated polymers with different protective agents in the pretreatment.

As shown in Figure 4, the products and yield distribution of lignin depolymerization were determined by GC–MS technology. The results indicate that the main products of degraded birch lignin include vanillin, methyl vanillate, 1-(4-hydroxy-3-methoxyphenyl) butan-1-one, eugenol aldehyde and other minor aromatic compounds. The detailed MS data were shown in Supplementary material (Figures S1–S5). Vanillin and eugenol aldehyde are essential raw materials in the food, pharmaceutical, and biomass industries. Other aromatic compounds derived from them serve as precursors for pharmaceuticals with antioxidant and antimicrobial properties, as well as building blocks for bio-based polymers and resins. These factors make them crucial to lignin valorization research. Compared to lignin without added protectants, the addition of protectants resulted in varying degrees of increase in the depolymerization product yield. Specifically, when formaldehyde was used as a protectant, there was a significant increase in the monomer yield of lignin, reaching 11.4% of the lignin addition, which is 2–3 times higher than that of lignin without protectants (4.7%). This may be attributed to formaldehyde pretreatment protecting more β-O-4 structures in birch lignin, which was confirmed by FT-IR, GPC, and 2D HSQC NMR characterization, demonstrating that formaldehyde facilitates the retention of the original lignin aromatic ether bond structure [42]. The distribution of lignin monomer yield indicates that the addition of protectants effectively protects the side chain C_α_-C_γ_ diol hydroxyl structure, reducing the degree of lignin polymerization to some extent and avoiding the formation of more difficult-to-degrade C–C bond structures, thereby promoting an increase in lignin monomer yield. Moreover, during lignin depolymerization, intermediate products undergo radical coupling reactions with methanol molecules under oxidative conditions to generate corresponding aldehyde and lipid compounds, thereby facilitating the conversion of lignin from macromolecules to aromatic compounds. Analyzing the distribution and yield of products is essential for elucidating changes in lignin structure and reaction pathways during depolymerization, providing guidance for lignin transformation into high-value chemicals.

**Figure 5:**
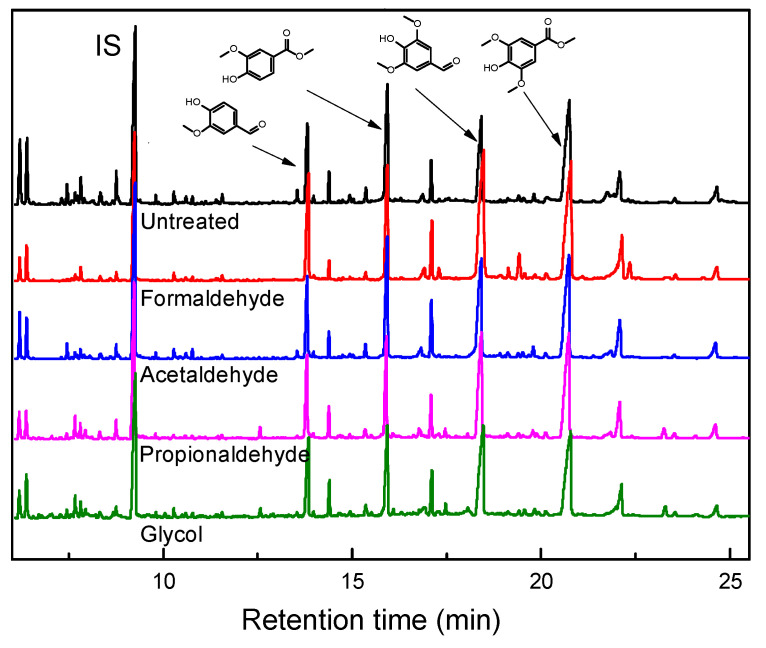
GC–MS spectra of lignin depolymerization products.

**Figure 6:**
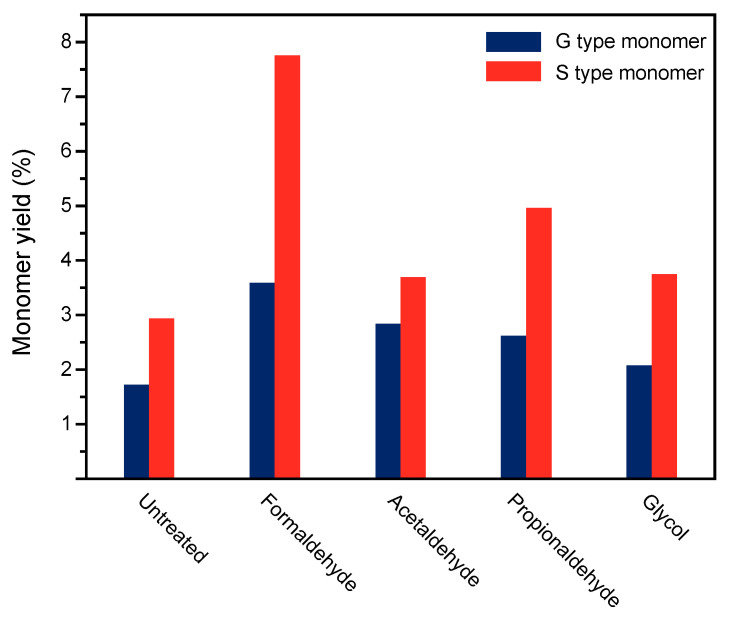
Monomer classification of lignin oxygenated polymers with different protective agents in pretreatment.

As depicted in Figure 5, the classification of monomer types resulting from the oxidative depolymerization of lignin treated with different protectants is shown. Combining the analysis with Figure 6, it is observed that the predominant products of birch lignin depolymerization are monophenolic compounds based on guaiacyl units (G-type) and syringyl units (S-type). This confirms the basic types of lignin structure in hardwood species, consistent with the notation in the aromatic region of lignin’s 2D HSQC NMR. For lignin depolymerization products under different protectant pretreatments, the yields of these two basic units after depolymerization have been effectively increased compared to untreated lignin. Particularly, formaldehyde treatment demonstrates a more effective protection of the diol structure on the syringyl units of lignin than on the guaiacyl units, resulting in a significant enhancement in the yield of S-type products in the depolymerization products.

### 3.3. The Effect of Pretreatment on the Transformation of Lignocellulose from Different Sources

**Figure 7:**
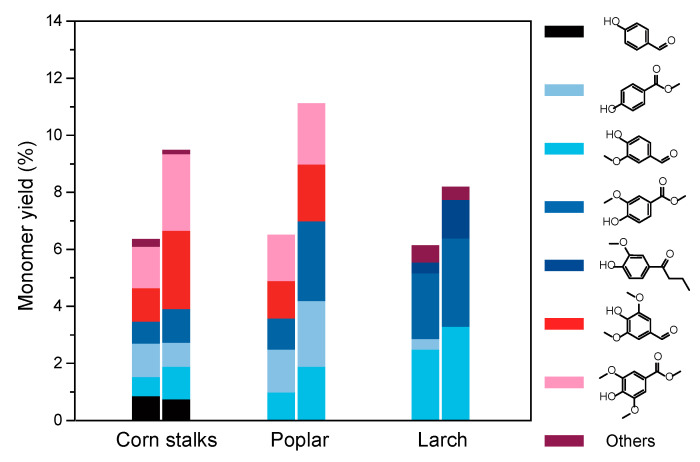
Effect of formaldehyde addition on lignin yield of corn straw, larch and poplar during pretreatment.

Based on the experiments above, it is evident that pretreating birch lignin with protective agents can effectively enhance the yield of aromatic compounds in the depolymerization products. To verify whether formaldehyde as a protective agent has a similar impact on the depolymerization yield of lignin from other sources, corn stover lignin, poplar wood lignin, and larch wood lignin were separately pretreated with formaldehyde and subjected to oxidative depolymerization. As shown in Figure 7, the yields of depolymerization products significantly increased after pretreatment with formaldehyde for corn stover lignin, poplar wood lignin, and larch wood lignin. Specifically, the monomer yield of corn stover lignin increased from 6.3% to 9.5% after pretreatment with formaldehyde; for poplar wood lignin, the monomer yield increased from 6.5% to 11.1%; and for larch wood lignin, the monomer yield increased from 6.1% to 8.2%. Moreover, regarding the distribution of products, corn stover, as a member of the herbaceous family, yielded products containing G, S, and H-type units. Poplar wood, as a broadleaf species, predominantly produced G and S-type units, with minor H-type units. Conversely, larch wood, a coniferous species, mainly yielded products with G-type basic units. These experimental results demonstrate that the protective strategy represented by formaldehyde has a similarly positive promoting effect on the oxidative depolymerization of lignin from different sources, providing valuable insights for the pretreatment of lignocellulose fibers.

### 3.4. Proposed Mechanism

**Figure 8:**
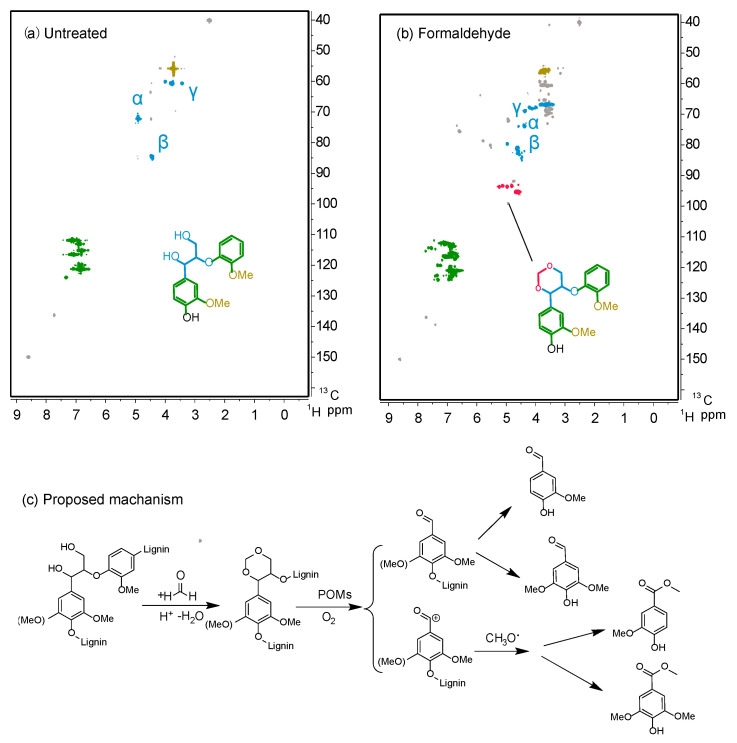
HSQC of lignin model compound before (**a**) and after reaction with formaldehyde (**b**), and (**c**) the proposed reaction mechanism.

During biomass pretreatment, the addition of protective agents, represented by formaldehyde, facilitates the extraction of lignin monomers. The primary challenge in developing this process lies in preventing interunit C–C coupling during extraction. As depicted in Figure 8a,b, firstly, in an acidic and dehydrated environment, formaldehyde reacts with the diol structures on the lignin side chains to form stable cyclic structures, inhibiting the formation of benzyl cations. This structure was confirmed using 2D HSQC NMR characterization of model compounds, as indicated by the red portion in Figure 8b. Secondly, the electron-rich positions adjacent to or ortho to the methoxy groups on the lignin aromatic ring are most reactive towards (protonated) formaldehyde, occupying these reactive sites and resulting in the formation of numerous ether linkages. During the reaction with POMs, the oxidative-reductive reaction occurs due to the strong acidity and oxidizing properties of POMs, leading to the cleavage of ether linkages in lignin. Some of these linkages are directly oxidized to aldehyde compounds like vanillin, while others undergo further oxidation and react with hydroxyl radicals in methanol to form ester compounds like methyl vanillate (Figure 8c).

## 4. Conclusions

Our investigation involved pretreating birch lignocellulose with formaldehyde, acetaldehyde, propionaldehyde, and ethylene glycol as protective solvents, aiming to inhibit the formation of recalcitrant carbon–carbon bonds during oxidative depolymerization. The objective was to enhance lignin conversion efficiency and boost aromatic compound yields in the resulting depolymerization products. Compared to untreated lignin, the pretreated lignin exhibited increased monomer yields following oxidative depolymerization. Notably, formaldehyde pretreatment significantly inhibited the hydrolysis of lignin aryl ether bonds, leading to a substantial increase in monomer yield under multi-acid catalysis, achieving up to 11.4% from birch wood. The depolymerization products comprised valuable aromatic compounds such as vanillin and syringaldehyde. Overall, the formaldehyde pretreatment strategy exhibited similar beneficial effects across various lignocellulosic biomass sources, enhancing monomer yields after oxidative depolymerization.

## Figures and Tables

**Table 1:** Distribution of lignin ash, water content, average molecular weight and average molecular weight after adding different protective agents.

Samples	Untreated	Formaldehyde	Acetaldehyde	Propionaldehyde	Glycol
*Mw* (g/mol)	7700	9600	11,300	7900	8000
*Mn* (g/mol)	3500	4300	5000	4000	4500
PDI	2.20	2.23	2.26	1.98	1.78

## Data Availability

Data are contained within the article and Supplementary Materials.

## Supplementary Materials

The following supporting information can be downloaded at: https://www.mdpi.com/article/10.3390/polym16243480/s1, Figure S1. The mass data of syringaldehyde; Figure S2. The mass data of methyl 3,5-dimethoxy-4-hydroxybenzoate; Figure S3. The mass data of methyl vanillate; Figure S4. The mass data of butyrovanillone; Figure S5. The mass data of vanillin; Table S1: The 2D HSQC NMR ^1^H-^13^C assignment of lignin aromatic and side chain area.
