# Supplementary material for: Transformation of Lignin Under Protection Strategies: Catalytic Oxidation and Depolymerization by Polyoxometalates Catalysts
Source: Polymers (Basel). 2024 Dec 13;16(24):3480. doi: 10.3390/polym16243480 (PMC11677733; doi:10.3390/polym16243480)
Supplement: Supplementary file 1 [file polymers-16-03480-s001.zip › polymers-3324530-supplementary.pdf]

Transformation of Lignin Under Protection Strategies: Catalytic Oxidation and Depolymerization by Polyoxometalates Catalysts

Xinyue Ma [1] and Wenbiao Xu [2,*]

[1] Faculty of Bioscience Engineering, Jilin Agricultural Science and Technology University, Hanlin Rond, Jilin City 132101, China;
[2] College of Materials Science and Engineering, Beihua University, Jilin City 132013, China;
* Correspondence: wenbiao.xu@beihua.edu.cn (W.X.)

Supplementary material

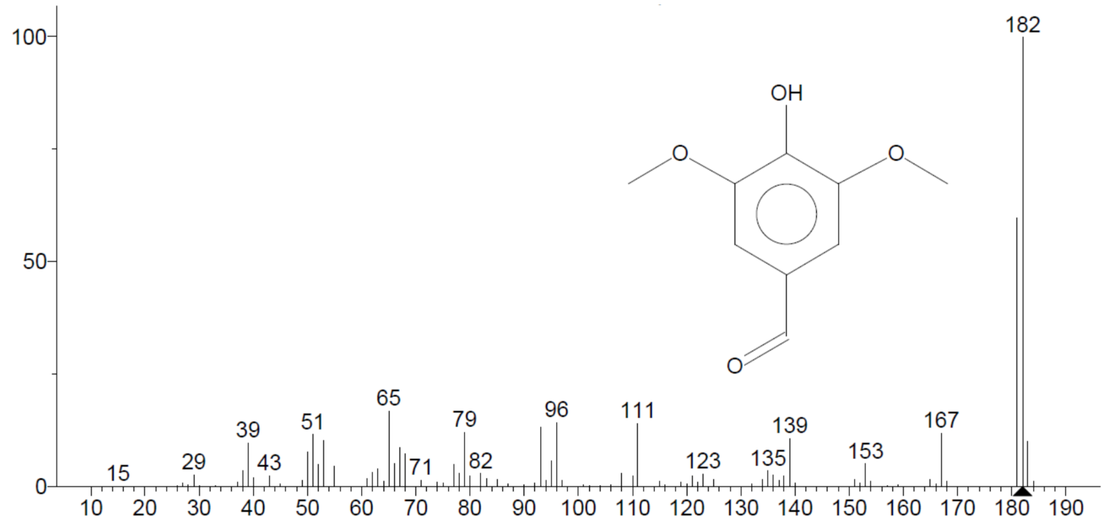

Figure S1. The mass data of syringaldehyde.

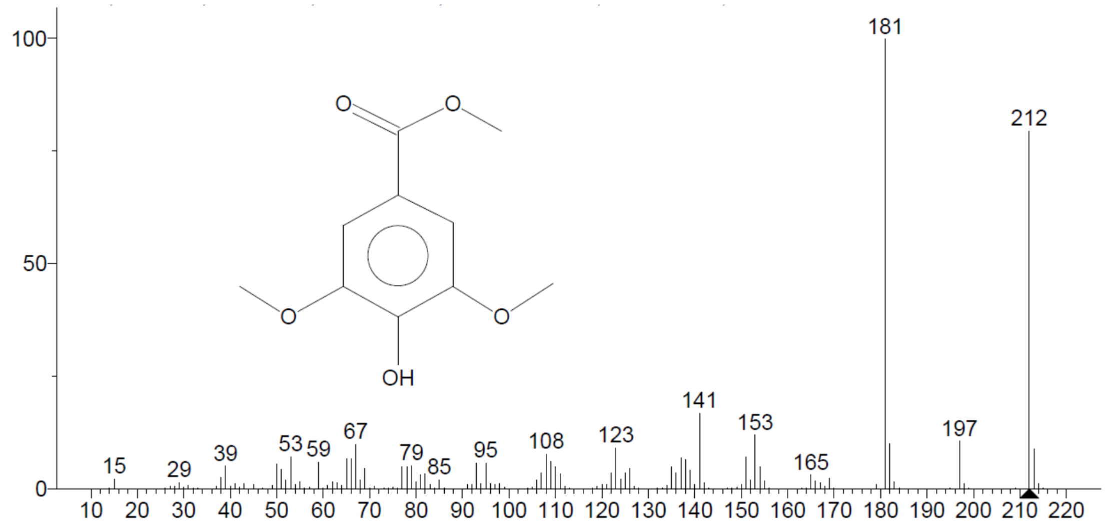

Figure S2. The mass data of methyl 3,5-dimethoxy-4-hydroxybenzoate.

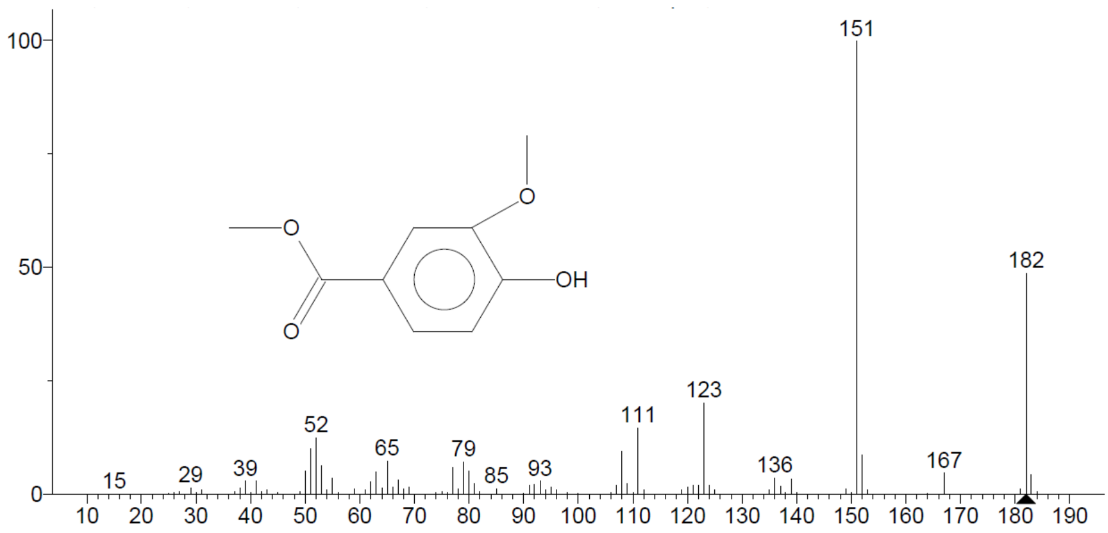

Figure S3. The mass data of methyl vanillate.

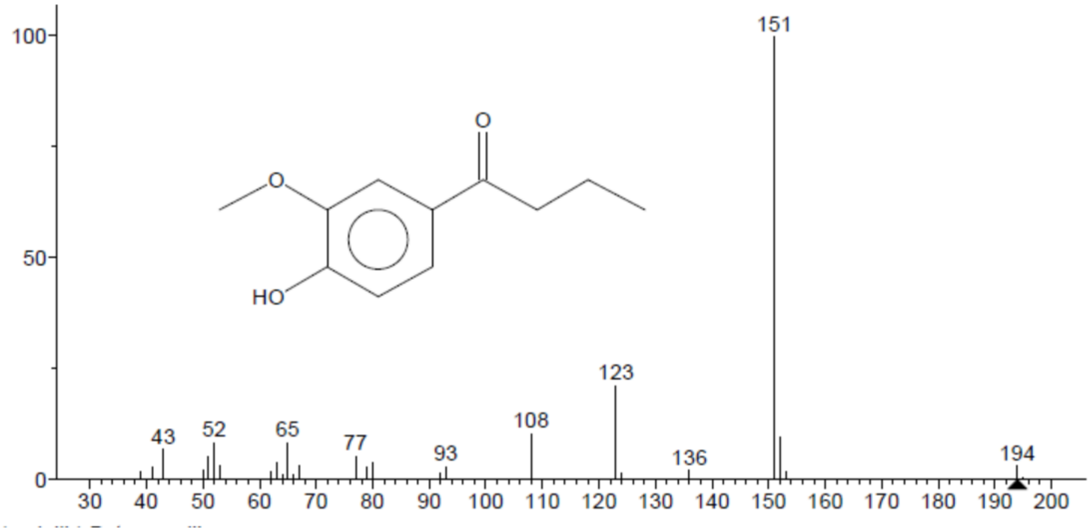

Figure S4. The mass data of butyrovanillone.

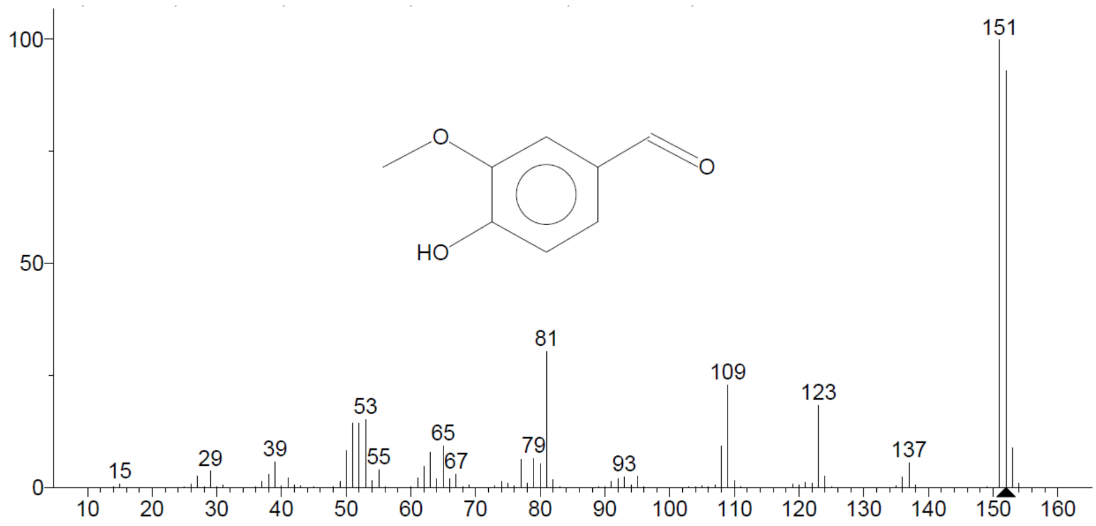

Figure S5. The mass data of vanillin.

Table. S1 The 2D HSQC NMR ^1H-^{13}C assignment of lignin aromatic and side chain area [1].

Label	δ_C/δ_H Chemical shift	Assignment
C_β	53.3/3.46	C_β-H_β in phenylcoumarane substructures (C)
B_β	53.5/3.06	C_β-H_β in resinol substructures (B)
$-OCH_3$	55.6/3.73	C-H in methoxyl groups
A_γ	59.5/3.40-3.61	C_γ-H_γ in β-O-4' substructures (A)
I_γ	61.4/4.10	C_γ-H_γ in *p*-hydroxylcinnamyl alcohol end- groups
C_γ	62.5/ 3.63	C_γ-H_γ in phenylcoumarane substructures (C)
A'_γ	63.2/3.83 and 4.30	C_γ-H_γ in g-acetylated β-O-4′ substructures (A')
A_α and A'_α	70.9/4.81	C_γ-H_γ in β-O-4' substructures (A) and g-acetylated β-O-4' substructures (A')
B_γ	71.0/3.61 and 4.18<br>71.0/3.61 and 4.18	C_γ-H_γ in resinol substructures (B)
B_α	84.8/4.65	C_α -H_α in resinol substructures (B)
A_α	85.9/4.10	C_α-H_α in β-O-4′ substructures (A)
$S_{2,6}$	103.8/6.71	$C_{2,6}$-$H_{2,6}$ in etherified syin gyl units (S)
$S'_{2,6}$	106.2/7.23	$C_{2,6}$-$H_{2,6}$ in oxidized (C_α = O) syin gyl units (S)
G_2	110.9/6.99	C_2-H_2 in guaiacyl units (G)
G_5 and G_6	114.9/6.72 and 6.94,<br>118.7/6.77	$C_{2,6}$-$H_{2,6}$ in oxidized (C_α = O) syin gyl units (S)

References

1. Lancefield, C. S.; Ojo, O. S.; Tran, F.; Westwood, N. J., Isolation of Functionalized Phenolic Monomers through Selective Oxidation and C-O Bond Cleavage of the β-O-4 Linkages in Lignin. *Angewandte Chemie International Edition* **2015,** 54, (1), 258-262.
